# Home‐based graded exposure to egg to treat egg allergy

**DOI:** 10.1002/clt2.12068

**Published:** 2021-10-12

**Authors:** Sarah Cotter, Dhanis Lad, Aideen Byrne, Jonathan O'B Hourihane

**Affiliations:** ^1^ Paediatrics & Child Health University College Cork Ireland; ^2^ Children's Health Ireland at Crumlin Dublin Ireland; ^3^ Paediatrics Trinity College Dublin Ireland; ^4^ Paediatrics and Child Health Royal College of Surgeons Dublin Ireland; ^5^ Children's Health Ireland Dublin Ireland

To the Editor,

Until recently, the standard treatment of egg allergy was complete avoidance of egg, awaiting natural acquisition of tolerance (71% by 6 years of age).^1^, ^2^ Recent studies have promoted oral tolerance by graded reintroduction of some allergenic foods.^3^ For egg allergy, this entails introducing egg in a stepwise fashion, from baked to almost raw. In many facilities worldwide, this method of treatment is implemented under the guidance of specialist clinicians or dietitians in clinic with a series of appointments to supervise progress at each step.

In Ireland, the minority of egg allergic children whose presenting reaction to egg resulted in life‐threatening anaphylaxis are not routinely offered home‐based tolerance induction programmes. The remaining children are offered home‐based tolerance induction, using the Irish Food Allergy Network (IFAN) guidelines for escalating exposure to egg, but based on home introduction without clinic visits, using the IFAN Egg Ladder as a guiding protocol.^4^ The IFAN Egg Ladder (Appendix 1) has varying degrees of cooked egg in food ranging from fully baked biscuits, to raw egg in meringue or mayonnaise. This visual aid is provided to parents along with an information leaflet, that advises families how to climb, stay or descend the Ladder, according to their child's tolerance of each exposure.^4^ The time period for each step is fluid, not fixed, as children do not acquire tolerance at a fixed rate.

Our clinical practice, in a resource limited setting, is that any infant with another index food allergy, usually milk or peanut, but without a parent report of safe consumption of egg or peanut, is skin tested for egg and peanut. If egg skin prick test (SPT) is positive then the egg ladder is started and depending on wheal size for peanut, supervised consumption or a formal food challenge with peanut is discussed with the family. Oral food challenges are not routinely performed at diagnosis in our service and it is acknowledged that not all SPT positive children are certain to be clinically reactive. The egg ladder is initiated immediately instead.

Families are advised in clinic how to treat allergic reactions with oral antihistamines and, if necessary (based on the clinical history), intramuscular adrenaline. Unlike formal immunotherapy programmes for peanut allergy,^5^ prescription and availability of adrenaline is not an absolute requirement to embark on the IFAN Egg Ladder‐based tolerance induction programme.

This report is a service evaluation through retrospective chart review. The data was collected from the paediatric allergy clinic, in Cork University Hospital. Ethical approval was granted from the Cork Research Ethics Committee of Cork Teaching Hospitals in February 2019 (EMC 6 (i) 12/02/19).

Twenty‐nine patients with confirmed egg allergy (*n* = 18, positive history and positive SPT and/or serum specific IgE [spIgE] to egg) or suspected/likely egg allergy (*n* = 11, no history of adverse reaction but positive SPT/spIgE to egg when presenting with another food allergy – usually milk or peanut) are reported, diagnosed by allergy‐focused clinical history taken by a single experienced allergist (JOBH), with universal SPT and occasional spIgE measurement, who were advised to use the IFAN Egg Ladder at home. Those recruited had been assessed and commenced on the IFAN programme in 2017 & 2018. Participant progress was followed to a cut‐off date of October 2019. The time in months from the initial intervention to tolerance of egg in the diet was measured.

The primary objectives were to examine degrees of tolerance to ingestion of egg obtained by 6 months and by 1 year after initial attendance. Achieving tolerance was defined as tolerating foods on step 3 of the egg ladder. Secondary outcomes were: (i) to discern whether the existence of concurrent peanut allergy, eczema or other food allergies have an effect on time to tolerance of egg after treatment. (ii) to clinically assess any possible barriers to the success of home‐based treatment. This was documented by the specialist Consultant during the assessment, if deemed significant. For the purposes of this study, a barrier was defined as a condition or circumstance that was considered to potentially impact on the progression of the stepwise protocol in the IFAN guidelines.

Twenty‐nine participants were followed, 19 (66%) were male and 10 (34%) were female. Baseline characteristics of the cohort can be seen in Table 1.

**TABLE 1:** Baseline demographic characteristics

	Followed in clinic	History of egg exposure	No history of egg exposure
	*n* = 29	*n* = 18	*n* = 11
At initial appt			
Age in months	Median 18	Median 17	Median 20.5
SPT wheal (mm)	Mean 3.8	Mean 4	Mean 3.6
SpIgE egg (KU_A_/L)	Mean: 12.4	Mean: 7.3	Mean: 14.5
	Range: 3.1–24.5	Range: 7–7.5	Range: 3.1–24.5
Index reactions			
Anaphylaxis	3	3	0
Skin reaction	14	14	0
GI reaction	1	1	0
Positive SPT, no exposure	11	0	11
Comorbidities			
Eczema	16 (55%)	12 (67%)	4 (36%)
Asthma	1 (3%)	0 (0%)	1 (9%)
Allergic rhinitis	2 (7%)	1 (6%)	1 (9%)
Peanut allergy	15 (52%)	8 (44%)	7 (64%)
Other food allergy	15 (52%)	7 (39%)	8 (73%)
Allergy profile			
Egg only	7 (24%)	5 (28%)	2 (18%)
Peanut & egg only	7 (24%)	5 (28%)	2 (18%)
Egg, peanut & other food allergy	8 (28%)	3 (17%)	5 (45%)
Egg & other food allergy but not peanut	7 (24%)	4 (22%)	3 (27%)
Medically perceived parental anxiety/reluctance to proceed with ladder	6 (21%)	3 (38%)	3 (27%)

The median time to tolerance for the 29 patients was 8 months. 12 (41%) children achieved this within 6 months and 20 (69%) children within 1 year of first appointment. These children were consuming and tolerating egg within step 3 of the ladder (at least scrambled egg) in their diet safely without allergic reaction. Only two patients made no progress on the ladder over 6 months and 1 year, both had parental reluctance reported as a barrier.

Participants with coexisting food allergy, other than peanut, on average, needed a significantly longer time period (11 m) compared to those without other food allergy (8 m *p* = 0.02) to achieve tolerance. See Figure 1. These allergies included milk, soya, legumes, kiwi, other nuts and fish. Eczema, asthma, allergic rhinitis and peanut allergy did not affect the rate of acquisition of tolerance to egg. SPT and spIgE levels measured at initial assessment showed no correlation to time to tolerance.

**FIGURE 1:**
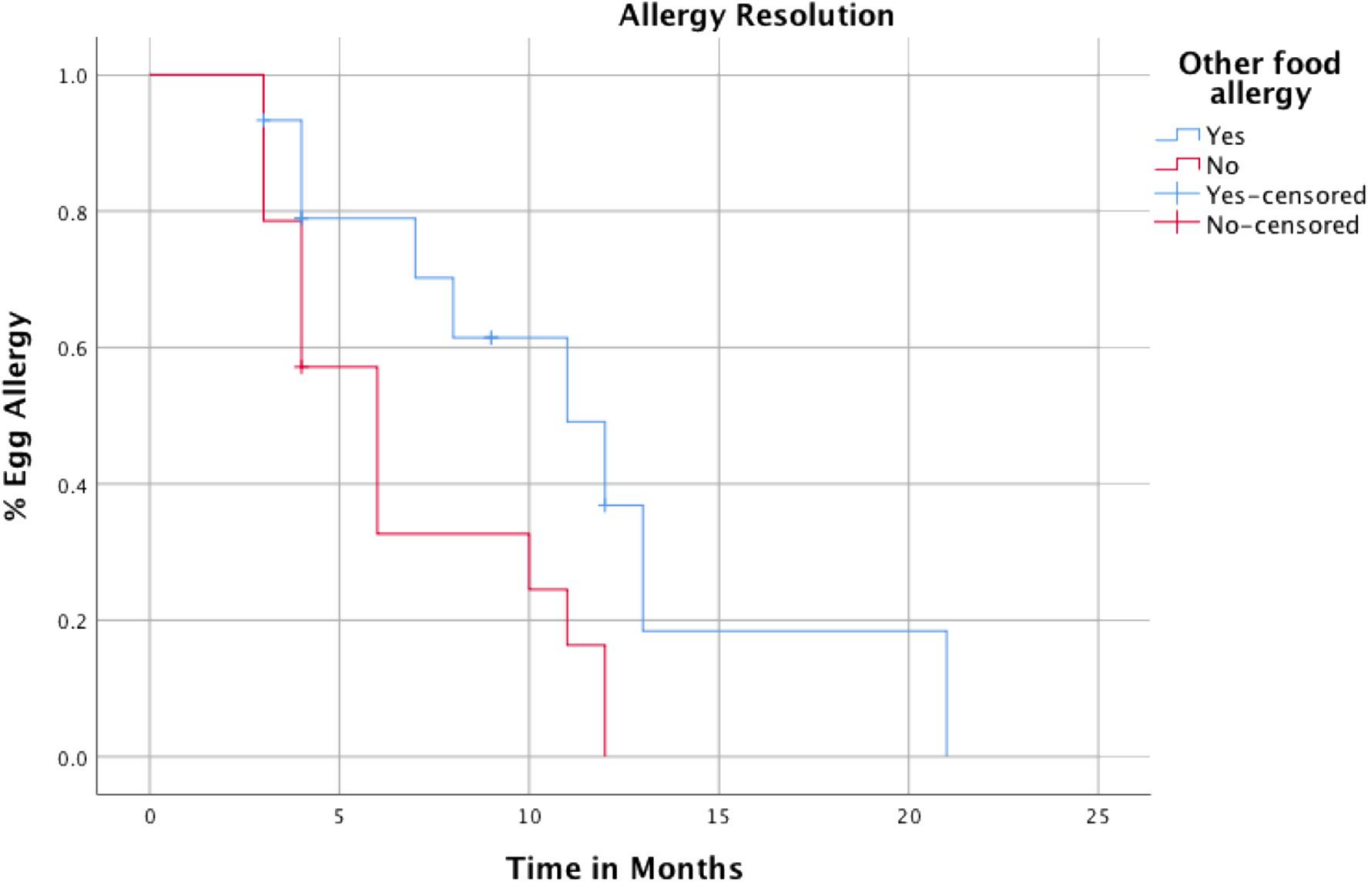
Time to tolerance according to presence of other food allergies (except peanut)

When assessing the ease with which the IFAN Egg Ladder was implemented in the home, medically identified parental anxiety was noted as a prominent barrier. However, this was a clinical assessment noted at the time and was not objectively measured. Reluctance to introduce egg at home, especially if the initial reaction was severe, was the most common theme seen.

One child (2%) reported anaphylaxis due to accidental ingestion of meringue, not because of planned escalation on the IFAN Egg Ladder. Adrenaline was correctly administered. The child subsequently achieved full tolerance within 4 months of the event.

The availability and opportunity of face‐to‐face interaction has become limited in the current climate of the Covid‐19 pandemic, creating a need for other safe and effective treatment methods. Outreach video and telephone clinics are becoming the new norm and we have recently shown they are safe for management of egg allergy in infants and can even start before face‐to‐face clinic attendance and testing.^6^

We consider these data show the IFAN Egg Ladder appears a safe and effective guide for treatment of most cases of egg allergy, implemented in the home environment, with remote, not in‐person support from an experienced allergy clinic and with individualised, not automatic prescription of adrenaline. We have practically eliminated the use of food challenges with egg from our clinical practice by using the egg ladder on an outpatient basis. Baked egg challenges are also very unusual now, reserved for children who have failed to progress at home in the first year of clinic attendance. Anecdotally these challenges are needed because of family anxiety about exposure and are usually passed. In our recent mass food challenge exercise^7^ only 10% of 27 egg and baked egg challenges were positive, compared to 80% of wheat and more than 50% of sesame challenges.

Children with both mild and severe egg allergy were able to establish tolerance in most cases, and it appears parental anxiety and reluctance to pursue the advised approach are significant barriers to progress. The amount of clinic time released with this supportive plan that works for most families could be used for the anxious families who cannot engage with the home egg ladder programme. As clinical practice moves to more virtual interfaces and support that appear very effective,^6^ all services must adapt safe existing and novel clinical programme to this new clinical environment. The IFAN Egg Ladder is suitable for this modernisation of clinical management of egg allergy.

## CONFLICT OF INTEREST STATEMENT

The authors declare no conflict of interests.

## Supporting information

Supplementary Material 1

Supplementary Material 2
