# Supplementary figures and images for: Home‐based graded exposure to egg to treat egg allergy
Source: Clin Transl Allergy. 2021 Oct 12;11(8):e12068. doi: 10.1002/clt2.12068 (PMC8506942; doi:10.1002/clt2.12068)

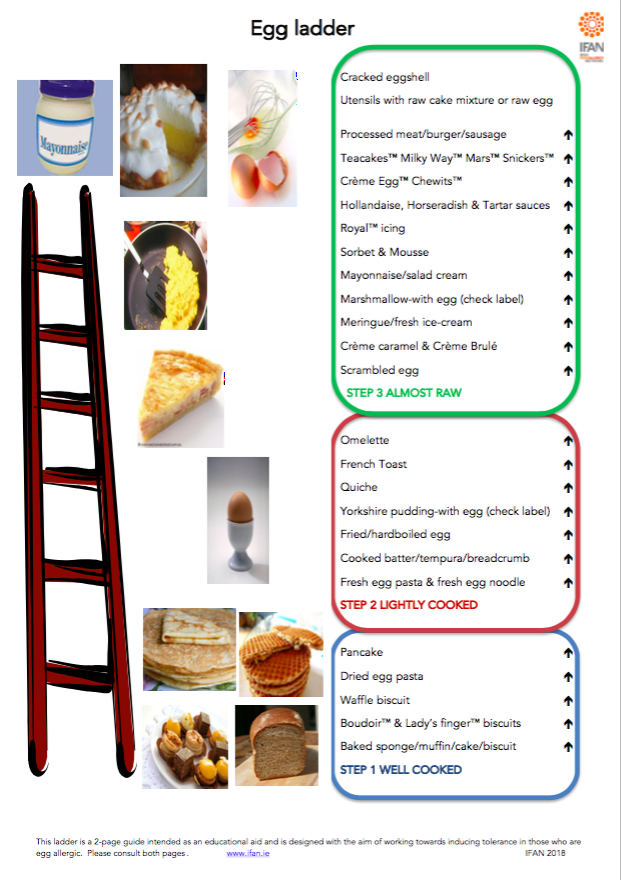

Supplement: Supplementary file 1 — Supplementary Material 1 [file CLT2-11-e12068-s001.png]

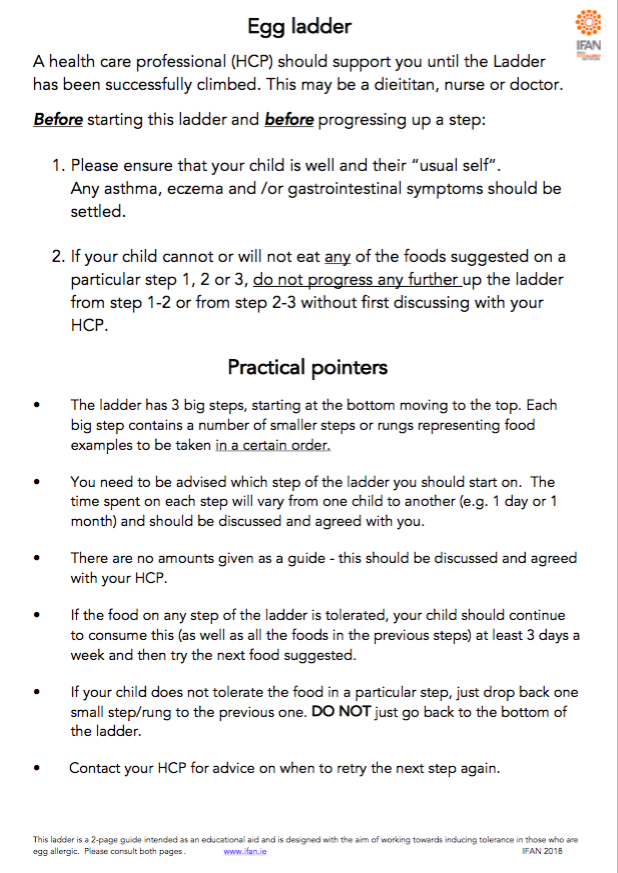

Supplement: Supplementary file 2 — Supplementary Material 2 [file CLT2-11-e12068-s002.png]
